# A Redox-Sensitive Micelle-Like Nanoparticle Self-Assembled from Amphiphilic Adriamycin-Human Serum Albumin Conjugates for Tumor Targeted Therapy

**DOI:** 10.1155/2015/987404

**Published:** 2015-05-13

**Authors:** Lin Chen, Feng Chen, Mengxin Zhao, Xiandi Zhu, Changhong Ke, Jiangming Yu, Zhiqiang Yan, Fulei Zhang, Yun Sun, Di Chen, Cheng Jiang, Xianxian Zhao, Yong Gao, Shangjing Guo, Wei Li

**Affiliations:** ^1^Department of Medical Oncology, East Hospital, Tongji University, 150 Jimo Road, Shanghai 200120, China; ^2^Department of Cardiology, Changhai Hospital, The Second Military Medical University, Shanghai 200433, China; ^3^International Joint Cancer Institute, The Second Military Medical University, Shanghai 200433, China; ^4^Department of Orthopaedics, Changzheng Hospital, The Second Military Medical University, No. 415 Fengyang Road, Shanghai 200003, China; ^5^Institute of Biomedical Engineering and Technology, Shanghai Engineering Research Center of Molecular Therapeutics, Shanghai 200062, China

## Abstract

The application of chemotherapeutic drug adriamycin (ADR) in cancer therapy is limited by its side effects like high toxicity and insolubility. Nanomedicine offers new hope for overcoming the shortcomings. But how to increase in vivo stability and to control intracellular drug release is a key issue for nano-based formulations. Herein, the hydrophobic ADR was successfully linked to the biocompatible human serum albumin (HSA) by disulfide bond 3-(2-pyridyldithio) propionyl hydrazide (PDPH), resulting in amphiphilic HSA-ADR. The novel ADR-HSA micellar NPs which were thus assembled exhibited a well-defined stable core shell structure with glutathione (GSH) sensitive linkers. The stable PDPH linkers at extracellular level were broken by GSH at intracellular level with a controlled ADR release profile. The in vitro cytotoxicity against gastric cancer cells (NCI-N87) was obviously enhanced by such redox-sensitive ADR-HSA NPs. Additionally, as observed by IVIS Lumina II Imaging System (Xenogen), the intratumor accumulation of ADR-HSA NPs was much higher than that of HSA/ADR NPs due to its high stability. Consequently, the in vivo tumor inhibition was significantly promoted after intravenous administration to the Balb/c nude mice bearing gastric tumors. These in vitro/vivo results indicated that disulfide-bond-containing ADR-HSA NPs were an effective nanodrug delivery system for cancer therapy.

## 1. Introduction

Adriamycin (ADR) is a kind of anthracyclines antitumor drug, which has strong cytotoxic effect and has been widely used in the treatment of liver cancer, lymphoma, gastric cancer, and breast cancer [1, 2]. But its low aqueous solubility and serious side effects such as bone marrow suppression, immunosuppression, and cardiac toxicity limited its clinical application [3, 4]. In order to reduce the side effects, nanoparticles such as liposomes, nanoparticles, and polymer micelles have been developed for improving the therapeutic effect [5, 6], among which, the new drug delivery system, HSA nanoparticles have received extensive attention due to its good biocompatibility, low toxicity and irritation, nonimmunogenicity, easy preparation, as well as the feasibility of drug targeting, sustained drug release and increased drug stability. HSA nanoparticles were regarded as a good drug carrier by fast accumulating in the rapid growing tumor tissues and delivering nutrition to cells with the intratumor drug release and improved the therapeutic effect [7, 8].

The HSA based formulation that was the paclitaxel (PTX) albumin nanoparticles solution (ABRAXANE) developed by American bioscience has been approved by the Food and Drug Administration (FDA) for clinical cancer therapy [9]. Clinical results showed that the HSA/PTX formulation was a potent antitumor drug. However, being an anticancer drug carrier material, HSA based formulations still created some problems to be solved including the low serum stability, easily to be broken, broad size distribution, and low intratumor accumulation of drugs, which was attributed to deformation of the physical blends [10]. The stability could be enhanced if the drugs and the HSA are linked together by chemical cross-linkers. The drugs and HSA linked by covalent bond can self-assemble into the micellar drug delivery system with stable structure and high tumor uptake efficiency [11, 12]. Besides, HSA is an ideal material for conjugating chemotherapy drugs, since it is a nutrient in the process of tumor proliferation [13, 14]. Therefore, the conjugation of antitumor drug (ADR) with HSA may result in reducing the systemic toxicity and increasing therapeutic effect of ADR.

It is known that the intracellular environment is different from extracellular environment which was characterized by the lower pH (4–6) and the redox states [15]. Intracellular glutathione (GSH/GSSG) concentration (0.5~10 mol/L) is over 200 times the extracellular GSH concentration (2~20 *μ*mol/L) [16]. The GSH in cells plays a crucial role for the redox condition regulation [17, 18]. In addition, compared with normal tissue cells, the tumor tissue cells have a more reduced environment due to their hypoxic states [19, 20]. All the above-mentioned concerning unique characteristics of intracellular conditions and tumor microenvironments offer new hope to screen a redox-sensitive bond for development of a reduction-sensitive drug carrier [21–23]. Among redox-sensitive bonds, the disulfide bond 3-(2-pyridyldithio) propionyl hydrazide (PDPH) is an ideal candidate. This PDPH can remain stable under the low concentration of GSH outside the cells. But it can be broken by the high concentration of GSH (intracellular level), resulting in the degradation of carrier, release of drug, and inhibition of tumor growth [24–28]. At present, the reduction sensitive bond is widely used in the drug/gene delivery system. And some new disulfide bonds were developed for controlled release recently [29].

In this study, the disulfide bond PDPH was utilized to link the ADR to the HSA, resulting in reduction-sensitive amphiphilic copolymer HSA-ADR. Then the novel micellar-like ADR-HSA nanoparticles were assembled by a rotary evaporation technique. The nanocarrier's properties, in vitro and in vivo antitumor effects were systemically investigated. This study provided an easy and feasible idea for the design and preparation of reduction-sensitive nanodrug delivery system.

## 2. Materials and Methods

### 2.1. Materials

Adriamycin was obtained from Dalian Meilun Biology Technology Co. 3-(2-Pyridyldithio) propionyl hydrazide (PDPH) was obtained from Pierce. HSA was obtained from Sigma with purity of 99%. 2-Iminothiolane hydrochloride (2IT) was purchased from Sigma. Dulbecco's modified Eagle's medium (DMEM) and Fetal bovine serum (FBS) were purchased from Gibco Co., USA. All chemicals were in analytic reagent grades.

### 2.2. Cells and Animals

The human stomach cancer NCI-N87 cell line, which was purchased from Shanghai Institute of Cell Biology, was cultured in DMEM supplemented with 10% FBS and 1% penicillin and streptomycin at 37°C with 5% CO_2_.

Male Balb/c mice (4–6 weeks, ~20 g) were obtained from Shanghai Experimental Animal Center of Chinese Academic of Sciences (Shanghai, China) and kept under SPF conditions. The animal experiments were performed in accordance with guidelines provided by the ethics committee of the Second Military Medical University, Shanghai, China.

### 2.3. Synthesis and Characterization of ADR-HSA

**Figure 1:**
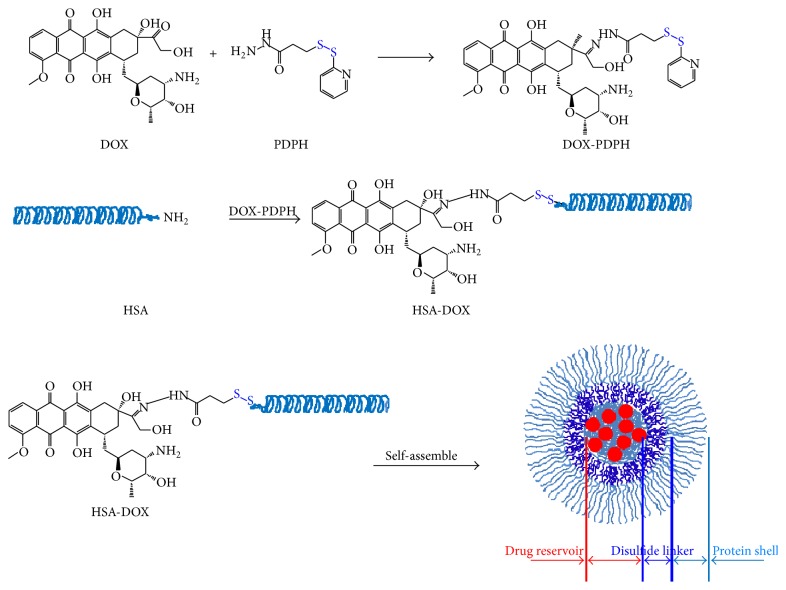
The scheme illustrated the synthesis and assembly process of ADR-HSA nanoparticles with well-defined structure.

ADR-HSA was synthesized according to the procedure shown in Figure 1. 2 mL adriamycin solution (4 mg/mL) was mixed with 2 mg PDPH and reacted in dark for a week. After reaction, the solution was dialyzed with a dialysis bag (MW cut off 3500) against dd water for 4 to 6 h to obtain the ADR-PDPH solution. The HSA was dissolved in dd water (2 mg/mL) and was thiolated through the reaction with 2IT. Then 2 mL ADR-PDPH solution (2 mg/mL) was mixed with 2 mL thiolated with molar ratio of ADR/HAS 50/1 and reacted for 2-3 h to obtain the ADR-HSA solution.

### 2.4. Preparation of ADR/HSA Nanoparticles (ADR/HSA NPs) and ADR-HSA Nanoparticles (ADR-HSA NPs)

In the experiments, 0.5 mg ADR-HSA was dissolved in 1.5 mL chloroform followed by drying under N_2_ atmosphere to form a thin film. Then the flask was connected with the equipment of rotation evaporation. The ADR-HSA film was eluted by 2 mL dd water; as a result, ADR/HSA NPs solution with concentration about 0.25 mg/mL was obtained. On the other hand, the ADR/HSA NPs solution were just prepared by mixing the drug ADR with HSA with the same concentration as ADR-HSA and dissolved in 2 mL dd water. For evaluation of cytotoxicity of the ADR-HSA nanoparticles (ADR-HSA NPs), the blend of the ADR/HSA nanoparticles (ADR/HSA NPs) at the same ADR concentration was served as control.

### 2.5. Size Distribution and Surface Potential of ADR-HSA NPs

The size distribution of ADR-HSA NPs was tested by the dynamic laser light scattering instrument (DLLS, ALV/CGS-3, Germany) at the scattering angle of 30°. The surface potential (*ζ*) of ADR/HSA and ADR-HSA NPs was tested by a Malvern Zetasizer 3000HS (Malvern, UK) using 5 mM NaCl as the baseline.

### 2.6. Transmission Electron Microscopy (TEM)

The morphology of particles was characterized by TEM and AFM. To prepare stained specimens for TEM (Hitachi, H-7000 Electron Microscope), PL-RB solution (0.5 mg/mL) was dropped on a 200-mesh Formvar-free carbon-coated copper grid (Ted Pella Type-A). After water evaporated, the sample was inversely covered on a small drop of 2% hydrodated phosphotungstate (PTA) solution. The conventional TEM images were obtained at 100 kV.

### 2.7. The In Vitro Release of ADR from NPs at Different GSH Concentrations

The in vitro drug release was conducted by the dialysis method. The ADR-HSA NPs solution in the membrane with concentration of 0.5 mg/mL was dialysed against 500 mL PBS at the pH ~7.3. At the predesigned time, about 0.05 mL ADR-HSA NPs solution was sampled and diluted by PBS to a volume of ~1 mL. At the same time, about 0.05 mL PBS was added to the ADR-HSA NPs solution inside membrane. For evaluating the effects of PDPH spacer on drug release, different concentrations of glutathione (GSH) were added to the drug solution. The ADR-HSA NPs solutions sampled at different time points were tested by the florescent at wavelength ~480 nm. The drug concentrations outside-/inside-membrane were calculated and the release profile was converted.

### 2.8. Cellular Uptake of ADR-HSA NPs by NCI-N87 Tumor Cells

The NCI-N87 cells in logarithmic growth phase were digested by 0.25% trypsin (containing 0.05% EDTA). The cells were resuspended in DMEM containing 10% FBS and inoculated on a glass bottomed petri dish, with 150 *μ*L per hole. The petri dish was then kept in a 5% CO_2_ incubator at 37°C overnight. The next day, the ADR/HSA NPs and ADR-HSA NPs suspension were diluted with DMEM containing 10% FBS (the concentration of ADR was 1 *μ*M). The culture medium was sucked out of the dish; then the sample solutions were added and incubated for 2 h at 37°C. The sample solution was removed, and the cells were washed using PBS solution twice and observed under laser scanning confocal microscopy (DMI4000 B, LEICA, Germany).

### 2.9. The Cytotoxicity of ADR-HSA NPs to NCI-N87 Tumor Cells

The CCK-8 method was used to determine the in vitro growth inhibition effect of ADR/HSA NPs and ADR-HSA NPs to tumor cells. The NCI-N87 cells were digested using 0.25% trypsin, inoculated in 96-well plates (corning, USA) with 2000 cells per hole, and cultured for 12 h. The samples of ADR/HSA NPs and ADR-HSA NPs were diluted to 6.25 nM to 200 nM (ADR). The culture medium in 96-well plates was removed and the different concentrations of samples were added and incubated for 12 h. The sample solutions were removed, and the CCK-8 solution was added. After 10 min, the absorbance was detected at 490 nm by enzyme-labeling instrument (PowerWave XS, Bio-TEK, USA). The percentage of surviving cells was calculated according to the following equation [30]:

(1) $$\begin{array}{c} \text{Surviving cells }\left(\;\%\;\right)=\frac{\left(A_{\text{sample}}-A_{\text{blank}}\right)}{\left(A_{\text{control}}-A_{\text{blank}}\right)}\times 100, \end{array}$$

where the *A*_sample_, *A*_blank_, and *A*_control_ are UV absorption at 485 nm from cell incubated with samples, the culture medium and the cell without samples.

### 2.10. In Vivo Tumor Accumulation Evaluation

Firstly, the Balb/c nude mice were inoculated subcutaneously on the right back with 5 × 10^6^ NCI-N87 cells (in 100 *μ*L culture medium) to develop xenografts tumor. After about 2 weeks, the volume of tumors reached about 50 mm^3^. For the in vivo distribution, mice bearing NCI-N87 tumor were randomly assigned to 3 groups with 3 mice/group (FICT, HSA/ADR-FITC, and HSA-ADR-FITC). Loading fluorescent isothiocyanate (FITC) HSA/ADR and HSA-ADR NPs with equivalent to 5 mg/kg dosage were administered via tail vein. 24 hours later, the tumors were viewed by IVIS Lumina II Imaging System (Xenogen), which was taken to capture the visible light photograph and luminescent image. Noted here, the mice with FITC administration at 0 hour were used as control. The in vivo images were observed with IVIS imaging system (excitation 500 nm) and recorded by a built-in CCD camera [31]. For further checking the tumor accumulation, the slides of the HSA/ADR and HSA-ADR NPS groups were anaesthetized by 1.5% isoflurane in 1 : 2 O_2_/N_2_. Mice were sacrificed after 24 h and the heart, liver, kidney, tumor, and spleen were excised. These organs were also imaged with the same excitation wavelength. Then the organs were collected and immediately fixed in formalin for 1 h. The organs were frozen in tissue-Tek-OCT and cryosections. Frozen sections were cut at 10 *μ*m and fixed with acetone at −20°C. After washing with PBS, sections were counterstained with 4′,6-diamidino-2-phenylindole dihydrochloride (DAPI; Sigma, Fluka Chemie, Buchs, Israel) and visualized by the confocal microscopy.

### 2.11. The In Vivo Antitumor Effect of ADR-HSA NPs

The nude mice model was constructed by injection of NCI-N87 cells (1 × 10^6^ cells in 200 *μ*L DMEM medium) subcutaneously. The nude mice were used for the experiment when the tumor tissue reached to 60 mm^3^. Three groups of nude mice models (*n* = 5) were intravenously injected with PBS (N.S.), ADR/HSA NPs, and ADR-HSA NPs, successively for 3 days. The dosage of ADR for each injection was 5 mg/kg, and the total dose of ADR for each group was 300 mg/mice. The volume of tumor tissue was measured every 2 days from the 1st day to the 33rd day. The tumor volume (*V*) was calculated according to the following equation [32]:

(2) $$\begin{array}{c} V_{\text{tumor}}=\frac{LW^{2}}{2}, \end{array}$$

where *L* and *W* were the longest and shortest diameters. The related tumor volume was calculated according to the formula: related tumor volume = Mean tumor volume at day recorded/mean tumor volume at day 0.

### 2.12. Statistical Analysis

Student's *t*-test was performed to measure the statistical differences of different groups (∗ for *p* < 0.05; ∗∗ for *p* < 0.01; ∗∗∗ for *p* < 0.001). The IC50 value was calculated by using the Prism ver5.02 program.

## 3. Results

### 3.1. Synthesis and Characterization of ADR-HSA NPs

**Figure 2:**
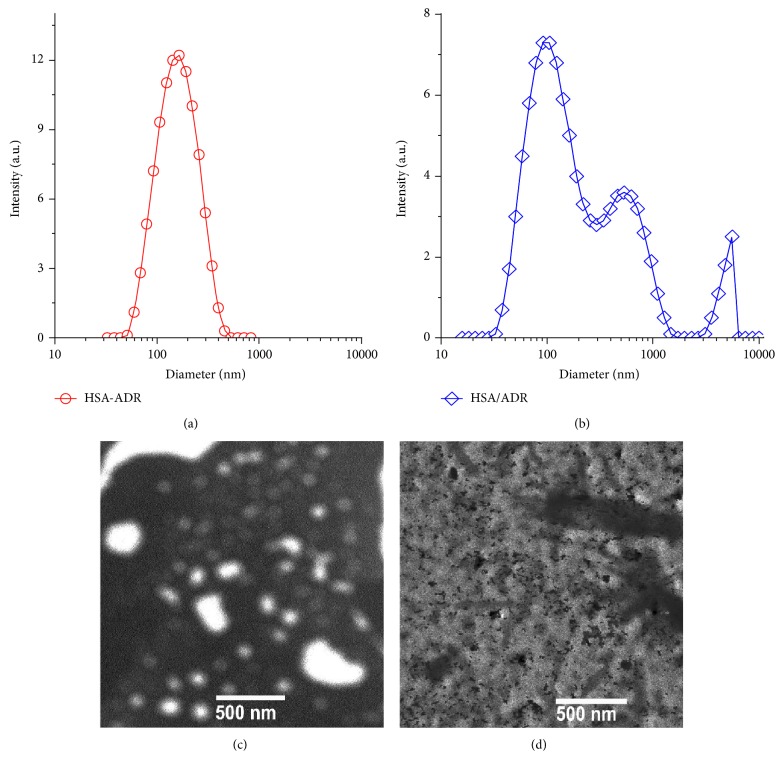
The size and size distribution of HSA-ADR (a), HSA/ADR (b); and the TEM photographs of HSA-ADR (c), HSA/ADR (d).

The chemical synthesis of protein-drug conjugates was shown in Figure 1; shortly, the ADR was modified by the disulfide bond (PDPH) firstly. Then the hydrophobic chemotherapeutic drug was linked to the HSA by the PDPH. Noted here, the average number of DOX on one HAS was about 15–20 as tested by UV. The well-defined core shell micellar-like HSA-ADR nanoparticles were self-assembled from the amphiphilic HSA-ADR by rotary evaporation method. The particle size and size distribution of ADR/HSA NPs and ADR-HSA NPs were measured by DLS method and the results were shown in Figures 2(a) and 2(b). ADR-HSA NPs exhibited a more narrowly distributed particle size, mainly around 100 nm (Figure 2(a)). The size of ADR/HSA NPs exhibited a multipeak distribution, mainly centered at 1000 nm (Figure 2(b)). The TEM photographs of ADR-HSA NPs and ADR/HSA NPs were shown in Figures 2(c) and 2(d). The ADR-HSA NPs exhibited nearly spherical shape with uniform size similar to that obtained by DLS. But it was hard to find some uniform particles for HSA/ADR system due to the large scale and unstable structure. These results suggested the superiority of the rotary evaporation and hydration technique. By contrast, it was hard to obtain spherical dispersed particles from the TEM images of ADR/HSA NPs (Figure 2(d)). The detailed properties of the HSA/ADR and HSA-ADR were summarized in Table 1.

### 3.2. In Vitro Release Profile of ADR

**Figure 3:**
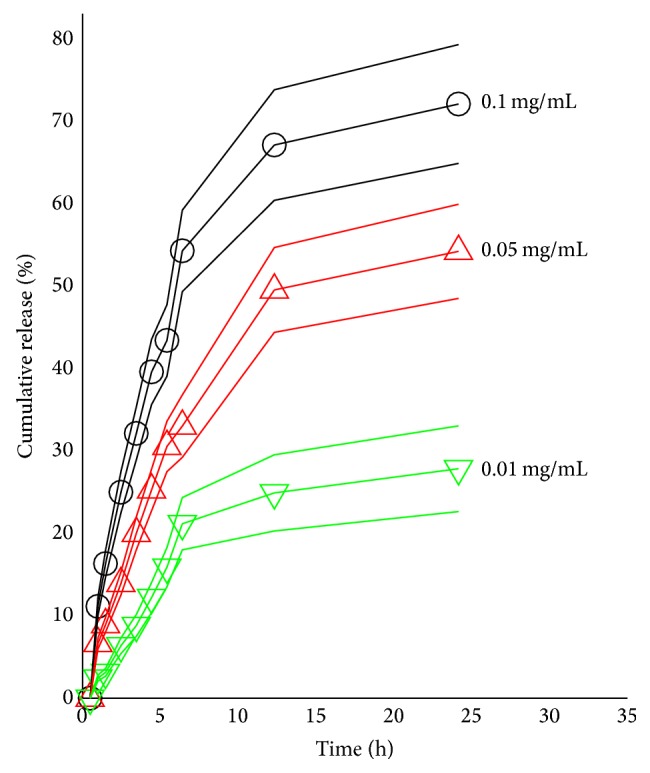
The in vitro drug release profile of HSA-ADR measured at different reducing agent GSH concentrations.

Figure 3 showed the glutathione (GSH) concentration dependence of the drug release profile. It was found that as the GSH concentration increased from 0.01 to 0.1 mg/mL, the cumulative released drug increased from 20% to 70% at the same time scale of 25 hours. There was an obvious concentration dependence of the ADR. As mentioned above, the GSH could break the PDPH linkers. So as the concentration of GSH increased, the PDPH breaking efficiency increased with more drugs released out.

### 3.3. Cellular Uptake of ADR-HSA NPs

**Figure 4:**
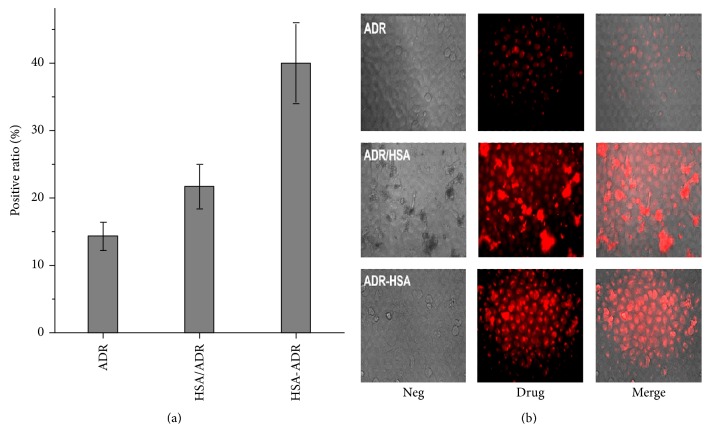
The fluorescent images of NCI-N87 tumor cells incubated with HSA, ADR, ADR/HSA NPs, and ADR-HSA NPs. The ADR/HSA NPs were aggregated and adsorbed on the bottom of petri dish, whereas the ADR-HSA NPs were internalized into the cell plasma and nucleus.

Figure 4(a) showed the cellular uptake profile of the ADR as transferred by ADR, HSA/ADR, and HSA-ADR. The positive ratio of HSA/ADR (20%) was higher than that of ADR group (15). The positive ratio of HSA-ADR was about 40%, which indicated that the cellular uptake of HSA-ADR nanoparticles was much higher than that of ADR and HSA/ADR groups. The differences of cellular uptake of ADR, ADR/HSA NPs, and ADR-HSA NPs were clearly indicated by the fluorescent images shown in Figure 4(b). The ADR can be internalized into the cell nucleus by the diffusion. For the ADR/HSA NPs group, we found some large drug-protein aggregates adsorbed on the bottom of the petri dish due to its instability, which lowered the internalization of ADR into the tumor cells. By contrast, ADR-HSA NPs can be internalized into the tumor cells with a high positive ratio. From the results, we could infer that once the ADR-HSA NPs were internalized into the tumor cell plasma, they would be degraded under the reduced circumstances, and the drug ADR would be released and entered the nucleus. Therefore, the results proved the reduction sensitivity of the ADR-HSA NPs.

### 3.4. In Vitro Cytotoxicity Evaluation of ADR-HSA NPs

**Figure 5:**
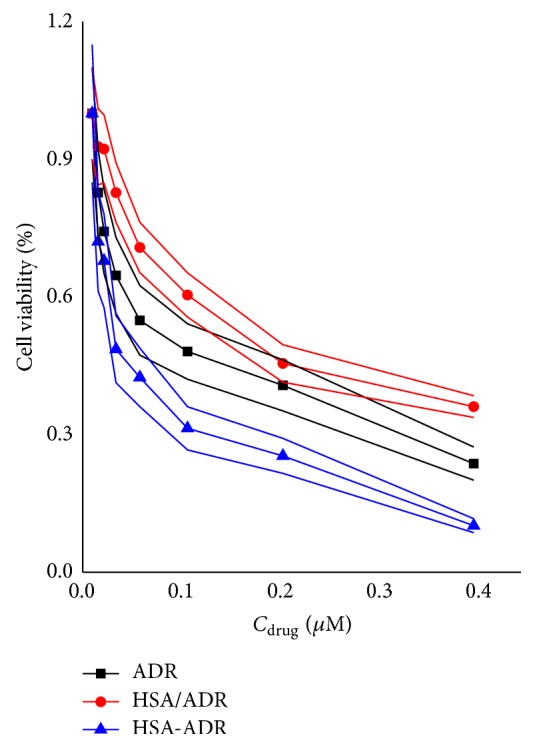
The in vitro cytotoxicity of ADR, ADR/HSA NPs, and ADR-HSA NPs to NCI-N87 tumor cells. ADR-HSA NPs showed a much lower IC50 value than ADR/HSA NPs, indicating the enhanced antitumor effect of ADR-HSA NPs compared with ADR/HSA NPs.

The in vitro cytotoxicity of ADR, ADR/HSA NPs, and ADR-HSA NPs against NCI-N87 tumor cells was shown in Figure 5. At the same drug concentration, the cytotoxicity trend was HSA/ADR < ADR < HSA-ADR. The IC50 (defined as half of the cells were killed) values for ADR, ADR/HSA NPs, and ADR-HSA NPs were 0.058, 0.165, and 0.031 *μ*M as summarized in Table 1, respectively. The IC50 for ADR/HSA NPs was about 5 times higher than that for ADR-HSA NPs, indicating the significantly enhanced cytotoxicity of ADR-HSA NPs to tumor cells. This result was in accord with the increased cellular uptake of ADR-HSA NPs compared with ADR/HSA NPs shown in Figure 4.

### 3.5. In Vivo Biodistribution and Antitumor Effect of ADR-HSA NPs

**Figure 6:**
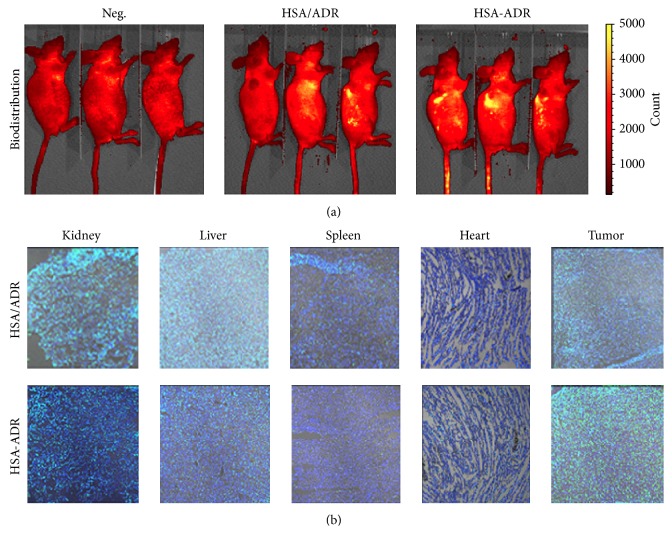
The biodistributions of Neg, ADR/HSA NPs, and ADR-HSA NPs illustrated by the living animal image (a) and the FITC accumulations in different organs' slides indicated by CLSM (b).

Figure 6 showed the in vivo biodistribution of the HSA/ADR and HSA-ADR nanoparticles monitored by the florescent molecule FITC. From the living animal's images (Figure 6(a)), it was easy to find that the intratumor accumulation of FITC delivered by HSA-ADR conjugated system was much higher than that of HSA/ADR blend system. This enhanced tumor accumulation of FITC was further confirmed by the slide images as shown in Figure 6(b).

**Figure 7:**
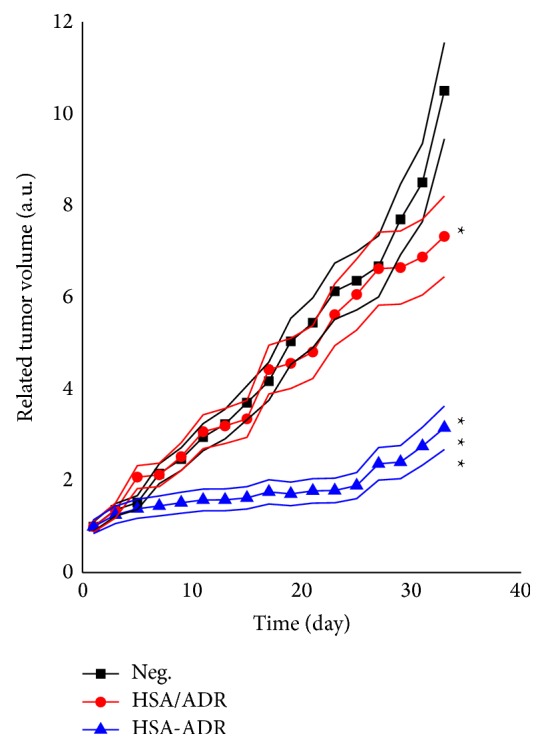
The inhibitory effect of N.S., ADR/HSA NPs, and ADR-HSA NPs shown as the relative tumor volume profiles. The antitumor effect of ADR-HSA NPs was much higher than that of ADR/HSA NPs.

The results of in vivo antitumor effects of ADR/HSA NPs and ADR-HSA NPs were shown in Figure 7. Compared with N.S., ADR/HSA NPs (*p* < 0.01) and ADR-HSA NPs (*p* < 0.001) showed a significantly more enhanced antitumor effect. Furthermore, the antitumor effect of ADR-HSA NPs was significantly higher than that of ADR/HSA NPs (*p* < 0.001). The tumor inhibitory rate of ADR/HSA NPs and ADR-HSA NPs was 22.91% and 69.98%, respectively. The enhanced inhibitory effect of ADR-HSA NPs should be in accord with their reduction sensitivity and the increased cellular uptake by tumor cells.

## 4. Discussion

It was known that the clinical application of small molecular chemotherapeutic drug adriamycin (ADR) was limited by its low solubility and high side effects. For overcoming the drawbacks, new nanoformulations based on liposomes, micelles were developed, among which, a successful sample was the HSA/PTX blend. This nanomedicine has been widely used in clinics. However, there still existed serious cytotoxicity such as bone marrow suppression, immunosuppression, and cardiac toxicity, which still strongly limited its clinic application. The limitation was attributed to the in vivo instability, drug leakage, and insufficient drug release. In this paper, we adopted a simple and feasible rotary evaporation and hydration technique to prepare novel reduction-sensitive nanoparticles ADR-HSA NPs. As evaluated by the UV and DLLS, the number of ADR conjugated on one HAS was about 15–20. As shown in Figure 1, the ADR-HSA NPs were successfully obtained by the rotary evaporation method. Its well-defined core shell structure and narrow size distribution were confirmed by the DLS and TEM as shown in Figure 2. To the best of our knowledge, this may be the first report on preparation of micellar-like HSA-ADR carriers with cleavable PDPH linkers.

In this new ADR-HSA NPs formulation, the outer shell was a biocompatible protein HSA. The inner core was composite of the hydrophobic drug ADR, while in the middle of the nanoparticles was a redox-sensitive linker PDPH, which can be degraded at a related high concentration of GSA, leading to the controlled drug release as shown in Figure 3. But it was stable when circulated in the blood due to the low GSH concentration. Additionally, compared with the HSA/ADR blends, the HSA-ADR NPs held the stable core shell structure with narrow distribution as confirmed by both Figures 1 and 2. All these properties showed that the HSA-ADR NPs were stable, which indicated that the ADR loaded in the micelles might not be leaked at the blood circulation. These characteristics could overcome the shortcomings like instability of traditional nanoformulation (HSA/PTX). The ADR-HSA NPs showed an increased cellular uptake with a high positive ratio shown in Figure 4 and an enhanced cytotoxicity shown in Figure 5.

**Figure 8:**
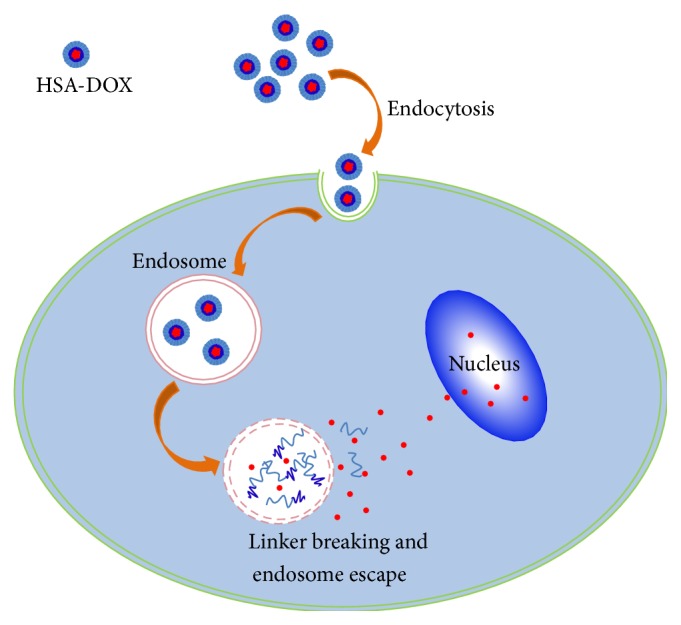
The scheme illustrated the mechanism of the cellular uptake and intracellular drug release of the HSA-ADR nanodrug delivery system.

The above-mentioned serum stability of the ADR-HSA NPs further enhanced the in vivo tumor accumulation as shown in Figure 6. This stable structure increased the interaction possibility of NPs with the tumor tissues [33]. The intratumor accumulation was thus obviously enhanced by the well-known EPR effects [34, 35]. On the intracellular level, the ADR-HSA NPs uptake was mainly dominated by endocytosis to form the endosome as shown in Figure 8. Noted here, the concentration of intracellular glutathione (GSH/GSSG) was about 0.5~10 mol/L, which was over 200 times the extracellular GSH concentration 2~20 *μ*mol/L. The PDPH linkers were thus remarkably degraded by such a high concentration of GSH, which resulted in the ADR release. Therefore, disulfide-contained drug carriers can timely release drugs dependent on the concentration of GSH in tumor cells. So compared with the HSA/ADR system, the ADR-HSA NPs significantly enhanced tumor growth inhibition as shown in Figure 7. In this study, we proved that the ADR can be rapidly released from the ADR-HSA under high GSH, thereby increasing the inhibitory effect to gastric tumors. Therefore, the disulfide-contained polymeric prodrug and nanoparticles are potential carrier systems for the treatment of tumors.

## 5. Conclusion

In summary, by a facile chemical conjugation of chemotherapeutic drug ADR to the biocompatible protein HSA, an amphiphilic molecule HSA-ADR was obtained with disulfide bond PDPH. Then the novel micellar-like ADR-HSA NPs were constructed by rotary evaporation method, which exhibited a well-defined core shell structure and GSH-sensitive releasing profile. The ADR release was controlled by the concentration of GSH. The in vitro cytotoxicity against NCI-N87 gastric cancer showed that such ADR-HSA NPs containing redox-sensitive PDPH linkers obviously enhanced the cellular uptake of ADR. In addition, the intratumor accumulation of such ADR-HSA NPs was much higher than that of the HSA/ADR blend due to its high serum stability and tumor accumulation. Consequently, the in vivo tumor inhibition by this ADR-HSA NPs was significantly promoted as intravenous administration to the Balb/c nude mice bearing NCI-N87 gastric tumor. These results indicated that disulfide-bond-containing ADR-HSA NPs were an effective nanodrug delivery system for targeting therapy of gastric cancer. This study may provide a new idea for the development of nanomedicine.

## Figures and Tables

**Table 1:** The effects of ADR, HSA/ADR, and HSA-ADR formulation composition on their in vitro/vivo performance.

	Size/nm	IC50/*μ*M	*ζ*/mV	Tumor inhibition rate (%)^∗^
ADR	0.5 ± 0.2	0.058	—	—
HSA/ADR	>1000 ± 250	0.165	~5–10	22.91
HSA-ADR	100 ± 10	0.031	~5–10	69.98

^∗^The tumor inhibition rate = (*V*_tumor  of  Neg._ − *V*_tumor  of  sample_)/*V*_tumor  of  Neg_.
